# Bridging the Digital Divide in Psychological Therapies: Observational Study of Engagement With the SlowMo Mobile App for Paranoia in Psychosis

**DOI:** 10.2196/29725

**Published:** 2022-07-01

**Authors:** Amy Hardy, Thomas Ward, Richard Emsley, Kathryn Greenwood, Daniel Freeman, David Fowler, Elizabeth Kuipers, Paul Bebbington, Philippa Garety

**Affiliations:** 1 Department of Psychology Institute of Psychiatry, Psychology & Neuroscience King's College London London United Kingdom; 2 South London & Maudsley National Health Services Foundation Trust London United Kingdom; 3 Department of Biostatistics and Health Informatics Institute of Psychiatry, Psychology & Neuroscience King's College London London United Kingdom; 4 Department of Psychology University of Sussex Brighton United Kingdom; 5 Department of Psychiatry University of Oxford Oxford United Kingdom; 6 Division of Psychiatry University College London London United Kingdom

**Keywords:** paranoia, psychosis, digital health, apps, human-centered design, user experience, adherence, engagement, therapy

## Abstract

**Background:**

Marginalized groups are more likely to experience problems with technology-related access, motivation, and skills. This is known as the “digital divide.” Technology-related exclusion is a potential barrier to the equitable implementation of digital health. SlowMo therapy was developed with an inclusive, human-centered design to optimize accessibility and bridge the “digital divide.” SlowMo is an effective, blended digital psychological therapy for paranoia in psychosis.

**Objective:**

This study explores the “digital divide” and mobile app engagement in the SlowMo randomized controlled trial.

**Methods:**

Digital literacy was assessed at baseline, and a multidimensional assessment of engagement (ie, adherence [via system analytics and self-report] and self-reported user experience) was conducted at 12 weeks after therapy. Engagement was investigated in relation to demographics (ie, gender, age, ethnicity, and paranoia severity).

**Results:**

Digital literacy data demonstrated that technology use and confidence were lower in Black people and older people (n=168). The engagement findings indicated that 80.7% (96/119) of therapy completers met the a priori analytics adherence criteria. However, analytics adherence did not differ by demographics. High rates of user experience were reported overall (overall score: mean 75%, SD 17.1%; n=82). No differences in user experience were found for ethnicity, age, or paranoia severity, although self-reported app use, enjoyment, and usefulness were higher in women than in men.

**Conclusions:**

This study identified technology-related inequalities related to age and ethnicity, which did not influence engagement with SlowMo, suggesting that the therapy design bridged the “digital divide.” Intervention design may moderate the influence of individual differences on engagement. We recommend the adoption of inclusive, human-centered design to reduce the impact of the “digital divide” on therapy outcomes.

**Trial Registration:**

ISRCTN Registry ISRCTN32448671; https://www.isrctn.com/ISRCTN32448671

## Introduction

Digital therapeutics have the potential to overcome barriers to the implementation of evidence-based health care, supported by the rapid growth in technology use. In the United Kingdom, approximately 79% of the population now own an internet-enabled mobile phone [1]. However, there is a well-documented “digital divide” whereby marginalized social, cultural, and demographic groups experience technology-related inequalities through the lack of access, confidence, and skills [2]. Given that engagement with digital therapeutics is a necessary condition for delivering benefit, reducing the impact of technology-related exclusion in minoritized groups is essential for equitable implementation [3,4]. This study therefore explored the “digital divide” and engagement in relation to SlowMo therapy, a blended digital therapy for paranoia in psychosis. SlowMo helps people learn to *slow down for a moment* to find ways of feeling safer. The technology consists of an intuitive web app to augment face-to-face individual cognitive behavioral therapy (CBT) sessions, which is synchronized with a native mobile app for use in daily life. In a recently completed randomized controlled trial (N=362), SlowMo demonstrated improved paranoia, self-concept, and well-being outcomes over 6 months compared with treatment as usual (TAU), with small to moderate effects [5]. This adjunct study investigated whether demographics commonly found to be associated with the “digital divide” were related to engagement in the SlowMo trial.

We propose that mental health is a highly relevant area in researching technology exclusion and health care engagement, as people in contact with mental health services have been found to be disproportionately affected by the “digital divide.” This is particularly marked in psychosis, with Robotham et al [6] reporting that approximately one-fifth of their sample was digitally excluded compared with only 3% of those with depression, although this rate had reduced from 30% in an earlier study [7]. Of note, excluded participants (ie, those with reduced technology access, confidence, and use) were significantly older—a finding that was replicated in a study examining factors associated with uptake of remote therapy in psychosis [8]. In a previous study by Robotham et al [7], Black people also had higher rates of exclusion, although this finding was not replicated in the follow-up study. A recent review found that White people and women with psychosis engage more with digital interventions than men and minoritized ethnic groups [9,10]. Given that the nature of technology use is complex and varies over time, there is a need for further research to investigate the “digital divide” in psychosis, especially as previous studies have tended to rely on small samples or purposive recruitment.

Perski et al [3] propose an evidence-based framework to account for how the “digital divide” may influence engagement, outlining that engagement is moderated by both the context (population and setting) and intervention (content and delivery). The emerging evidence indicates older, female, White, digitally skilled, and confident people without mental health problems appear more likely to engage, although further research is needed. The multidimensional assessment of engagement is also recommended, incorporating self-report and objective metrices [3]. This study used both experiential and behavioral assessments of engagement. A review of studies evaluating the usage of digital therapies in mental health found that more frequent and prolonged use was assumed to be desirable [11,12]. However, this assumption risks conflating engagement with adherence and not recognizing that disengagement may reflect the e-attainment of personal goals if skills acquisition has been sufficiently supported. It is therefore suggested that optimal usage should be defined a priori, based on the intervention’s theoretical principles and mechanisms of change.

The identified relationships between engagement, population characteristics, and intervention content and delivery underscore the need for digital interventions to be designed so that the widest range of people are willing and able to use them. Human-centered design is increasingly employed in health care innovation to enhance user experience, thereby promoting engagement [13-17]. SlowMo therapy is an exemplar of an inclusive, human-centered design approach and therefore aims to overcome barriers to implementation across diverse groups [18,19]. Our multidisciplinary team of experts by experience, clinicians, researchers, industrial designers, and software developers integrated best practice principles of design thinking and participatory design to co-design the therapy. A risk inherent in participatory design is that the most willing, able, and vocal users are more likely to be involved, neglecting the needs of minoritized groups. To address this, we purposively sampled people from a wide range of backgrounds (ie, gender, age, ethnicity, cognitive abilities, use of technology, and attitudes to therapy) [20,21]. Adopting design thinking methodology meant we were able to address the problem of digital solutions often being skeuomorphic, replicating analogue versions of therapy artefacts (eg, a pen-and-paper form for monitoring and evaluating thoughts) and therefore failing to address barriers to use [22,23]. Our design research identified the importance of SlowMo therapy being usable, trustworthy, enjoyable, personalized, and normalizing and of it offering flexible interpersonal support [19].

In summary, this study examined digital literacy and engagement in the SlowMo therapy trial sample [5] to investigate if there was evidence of a “digital divide” and if demographics were associated with engagement. The therapy sample was first characterized in relation to their digital literacy at baseline, followed by a description of the SlowMo mobile app adherence based on self-report and system analytics data, and user experience evaluated using a self-report questionnaire. Associations between demographic factors, digital literacy, and engagement were also investigated. The research questions were as follows:

1. What is the digital literacy of the therapy sample and is this associated with demographic factors (ie, gender, age, ethnicity, and paranoia severity), suggesting a “digital divide”?
2. Does the SlowMo mobile app demonstrate acceptable rates of self-reported and system analytics adherence, and is adherence associated with demographic factors (ie, age, gender, ethnicity, and paranoia severity)?
3. What are the self-reported rates of usefulness, enjoyment, and usability for the SlowMo mobile app, and is user experience associated with demographic factors (ie, age, gender, ethnicity, and paranoia severity)?

## Methods

### Design

This was a planned adjunct study to the SlowMo trial, a parallel-group randomized controlled trial that tested the efficacy of SlowMo therapy in reducing paranoia severity when added to TAU, compared with TAU, with 1:1 allocation and blinded assessors. Recruitment was from community mental health services with identical procedures across 3 main sites in England: South London and Maudsley National Health Service (NHS) Foundation Trust, Sussex Partnership NHS Foundation Trust, and Oxford Health NHS Foundation Trust. Additional patient identification centers, NHS trusts near each of the 3 main recruitment sites, were also used.

### Ethics Approval

The trial received a favorable ethical opinion (Camberwell St. Giles Research Ethics Committee: 16/LO/1862; IRAS: 206680).

### Participants

Inclusion criteria were an age of ≥18 years; persistent (≥3 months) distressing paranoia (assessed using the Schedules for Clinical Assessment in Neuropsychiatry [24]); a score of >29 on the Green Paranoid Thoughts Scale (GPTS), part B, persecutory subscale [25]; a diagnosis of schizophrenia-spectrum psychosis (F20-29, ICD-1025); capacity to provide informed consent; or a sufficient grasp of English to participate in trial processes. Exclusion criteria were profound visual or hearing impairment; the inability to engage in assessments; currently in receipt of psychological therapy for paranoia; or a primary diagnosis of substance abuse disorder, personality disorder, organic syndrome, or learning disability. All participants gave written informed consent. The primary outcome was self-reported paranoia severity measured by the GPTS over 24 weeks. From May 1, 2017, until May 14, 2019, we assessed 604 people for eligibility and, of these, recruited 362 participants: 181 were allocated to the SlowMo group, and 181 were allocated to the control group. Of the 181 participants in the SlowMo group, 168 (92.8%) engaged with at least 1 SlowMo therapy session, and 145 (145/181, 80.1%) completed all 8 sessions. The sample attending at least 1 session (n=168) were predominantly male (122/168, 72.6%), with a mean age of 42.77 (SD 11.99) years, and were mainly White British (111/168, 66.1%; followed by Black African: 13/168, 7.7%; Black Other: 13/168, 7.7%; Black Caribbean: 8/168, 4.8%; Asian: 6/168, 3.6%; mixed heritage not specified: 6/168, 3.6%; Arab: 5/168, 3.0%; Chinese: 4/168, 2.4%; Hispanic: 2/168, 1.2%). A schizophrenia diagnosis was most common (105/168, 62.5%; schizoaffective disorder: 23/168, 13.7%; other schizophrenia-spectrum diagnoses: 40/168, 23.8%).

Digital literacy was assessed at the beginning of therapy, with 91.1% (153/168) of participants providing data. System analytics adherence data incurred some data loss at the beginning of the trial due to a bug in the code. Once rectified, analytics data were stored when the participant had the version of the mobile app with the analytics code installed; for individuals in therapy when the analytics issue was resolved, the app could be updated to this version at any stage of therapy. Participants were defined as having missing analytics (28/168, 16.7%) when there were insufficient data points to determine mobile app adherence according to our a priori criteria of at least 1 home screen view for at least three sessions. Analytics adherence data were available for 83.3% (140/168) of those attending at least 1 session. Self-reported adherence and user experience surveys (UESs) were assessed at the end of therapy, so data were only available for participants who completed every therapy session. Further, this assessment was not offered to the first 45 therapy cases, and a further 3 participants were not eligible to complete the UES, as they declined any engagement with the SlowMo mobile app. User experience data were obtained for 83 participants (83/168, 49% of therapy attenders; 83/97, 85% completion rate once UES collection commenced).

### Intervention Structure, Content, and Technology

SlowMo consisted of 8 individual, face-to-face sessions, each module addressing a specific topic, typically lasting 60 minutes to 90 minutes, within a 12-week time frame. The intervention followed a clinical trial manual that was consistent across the trial. The software includes an intuitive web app to augment face-to-face individual therapy sessions, which is synchronized with a native mobile app for use in daily life. Therapy sessions were delivered at locations of the participants’ choosing, including clinic settings or at home, and behavioral work was carried out in the participants’ locality. Therapy was delivered by 11 trained doctoral-level psychologists (10 clinical and 1 counseling) experienced in CBT for psychosis, with weekly group supervision, using recorded sessions.

The SlowMo web app is delivered via a touch screen laptop with interactive features including information, animated vignettes, games, and personalized content. In sessions, people learn that fast thinking is part of human nature. However, fast thinking can fuel worries, and thinking slowly is helpful in dealing with fears about other people. This key principle frames the sessions in which people are supported to try out ways to slow down (eg, by considering the impact of mood and past experiences and looking for safer alternative explanations). SlowMo therapy is presented as a journey that supports people to notice the large, fast spinning, and grey worry bubbles that fuel distress and make use of slow spinning and colored bubbles to shrink fears and feel safer. The use of personalization, ambient information, and particularly the use of visual rather than verbal metaphors targeted a step change in therapy delivery by enhancing appeal and reducing cognitive demands.

The SlowMo native mobile app was installed on a standard Android smartphone provided to participants. It assists people to notice their fears and thinking habits as they occur in daily life and to access SlowMo strategies or personalized safer alternative thought bubbles. It consisted of a redesigned CBT thought record for managing paranoia, which is a commonly used tool for identifying and modifying distressing cognitions. Thought records are often digitally reproduced with the same interface as in paper versions, usually text prompts and response boxes presented as a form. These skeuomorphic designs do not address obstacles to the use of thought records, such as being cognitively demanding and having an unappealing, impractical interface. The mobile app interface therefore attempted to overcome the limitations of paper and skeuomorphic digital thought records. This incorporated an attractive visual representation of thoughts and their attributes (eg, conviction, distress, and thinking style); simple touch screen interactions to support monitoring and modifying thoughts; easy access to previously identified helpful suggestions and thoughts; positive reinforcement for engaging in slowing down; and a flexible interface that afforded several ways of slowing down fast thinking, depending on a person’s needs and preferences (eg, quick access to safer thoughts on the home screen or sequentially slowing down a thought over multiple screens). Concerns about privacy were addressed by developing a native app with opt-in data transfer. The mobile app also relied on user-initiated interaction and optional push notifications to accommodate those who might find notifications intrusive [26,27].

### Research Question 1: Digital Divide—Digital Literacy

This was assessed in relation to (1) the self-reported ownership of smartphones or access to a computer, (2) the frequency of use of smartphones (excluding phone calls) and computers, and (3) confidence in using smartphones and computers. The frequency of use and confidence were assessed on scales from 0 to 100, with the anchors of “never” to “all the time” and “not at all” to “totally,” respectively.

### Research Question 2: Engagement—System Analytics and Self-reported Adherence

System analytics adherence was operationalized as at least 1 out-of-session interaction for a minimum of 3 out of 7 possible therapy sessions (session 8 data could not be included, as mobile app data syncing did not occur following the end of therapy). The adherence criterion was based on the assumption that engagement with the mobile app would be indicative of its usefulness, usability, and appeal, with app use also potentially reducing as the skill of slowing down is internalized, reflecting e-attainment [11,28]. Home screen views were selected as the target interaction, given the multiple routes to slowing down can be accessed via the home screen. Self-reported adherence was assessed by asking participants how much they were using the mobile app and if they intended to use it in the future (rated from “0 – never” to “100 – all the time”).

### Research Question 3: Engagement—UES

A 12-item self-report measure of user experience with the mobile app was developed (see Multimedia Appendix 1), adapted from a 26-item self-report measure employed by [10]. The UES consisted of 4 items assessing usefulness, 4 items assessing usability, and 4 items assessing enjoyment. Each item was rated on a scale from 0 to 100, with anchors of “totally disagree” to “totally agree.” Items were summed (with 4 items reverse scored; range from 0 to 400 for each category), and a percentage score was calculated.

### Statistical Analysis

Summary statistics were calculated for all variables for the SlowMo therapy arm and split by site. The analysis investigated the associations between demographics and digital literacy (to evaluate the “digital divide,” research question 1) and demographics and engagement (assessed by behavioral [adherence] and experiential [self-reported user experience] metrices, research questions 2 and 3). Independent group *t* tests (gender and GPTS paranoia severity) or one-way ANOVAs (ethnicity and age) were performed for the continuous dependent variables of digital literacy, self-reported app adherence, and the UES, and chi-square tests were performed for smartphone ownership, computer access, and system analytics app adherence (rated adherent or nonadherent). Independent group *t* tests were also conducted to examine the association between system analytics adherence and pretherapy smartphone literacy. Categories for the participant demographics were gender (male, female), age (<35, 35-50, and ≥50 years), ethnicity (White ethnicity, Black ethnicity, and other minoritized ethnic groups), and paranoia severity (low and high, dichotomized by a median split of <61 and ≥62 on the GPTS). All statistical tests were 2-tailed

## Results

### Research Question 1: Digital Literacy and the Digital Divide

**Figure 1:**
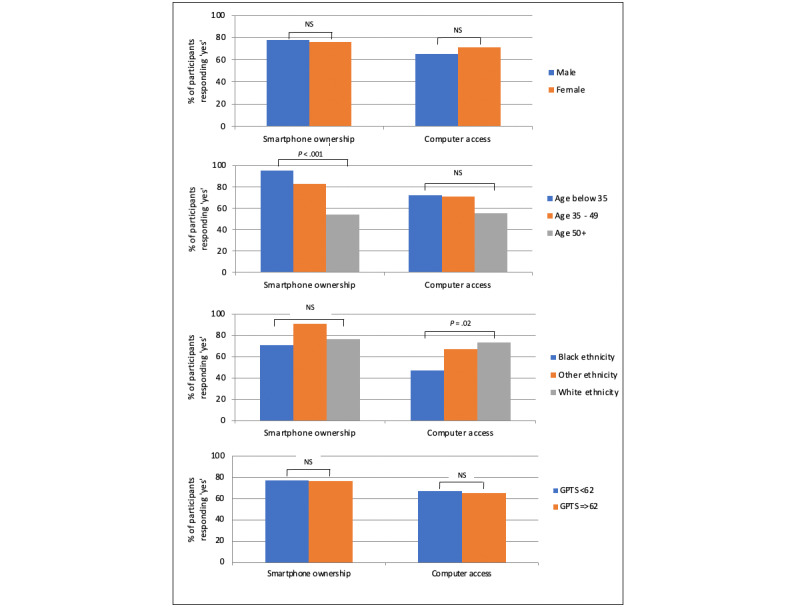
Smartphone and computer ownership by gender, age, ethnicity, and paranoia severity in people attending at least 1 therapy session (n=168). GPTS: Green Paranoid Thoughts Scale; NS: nonsignificant.

**Figure 2:**
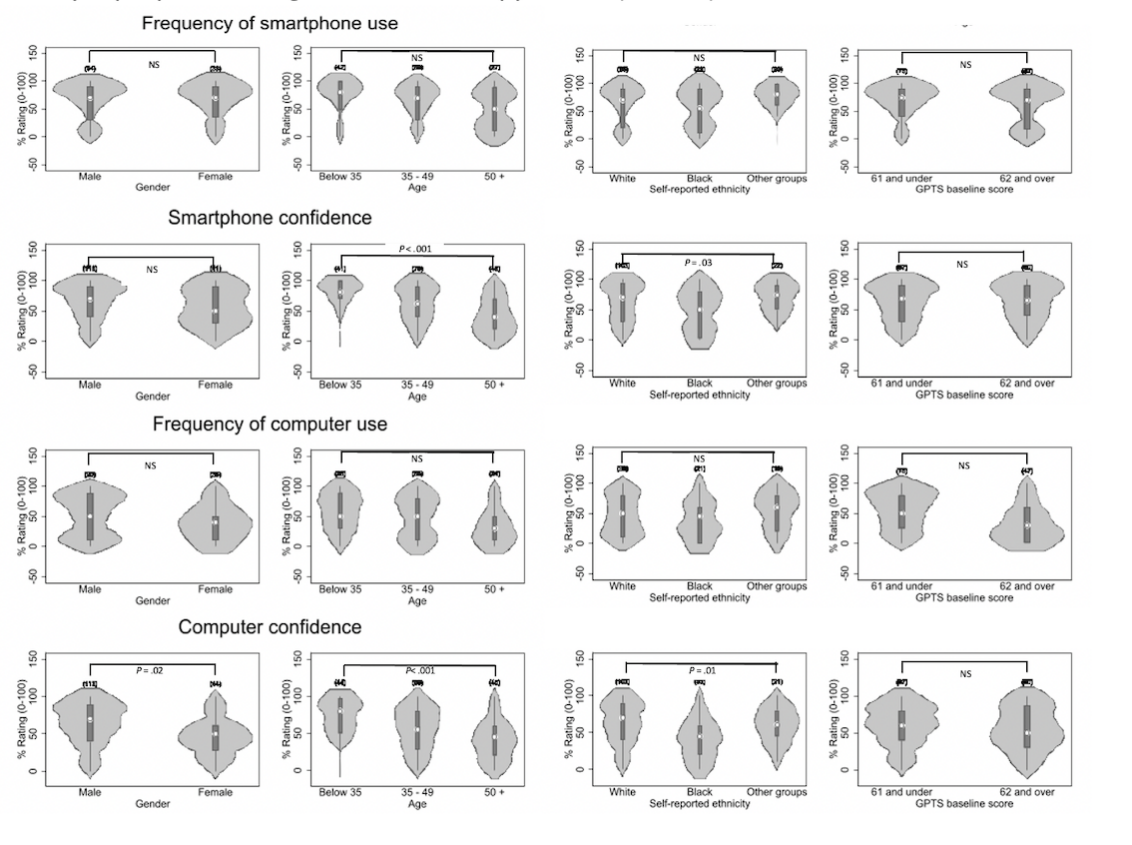
Frequency of smartphone use, smartphone confidence, frequency of computer use, and computer confidence by gender, age, ethnicity, and paranoia severity in people attending at least 1 therapy session (n=168). GPTS: Green Paranoid Thoughts Scale; NS: nonsignificant.

The SlowMo therapy group’s rates of smartphone ownership, computer access, technology use, and digital confidence are displayed in Table 1, by site and overall. This indicates that just over three-quarters of the sample owned a smartphone, which was consistent across all sites. The pattern of results suggests that computer and smartphone access, frequency of use, and confidence were generally lower in the inner-city site (London) compared with the other 2 sites, which were more rural (Oxford and Sussex). The impact of gender, age, ethnicity, and paranoia severity on smartphone and computer ownership, use, and confidence are shown in Figures 1 and 2, respectively, with inferential statistics presented in Table S1 in Multimedia Appendix 2. Ownership is a binary outcome and is represented using bar charts. Use and confidence are continuous variables and shown with violin plots, as these indicate the data distribution. In support of previous findings indicating a “digital divide” in relation to age, there were significant age differences in smartphone literacy, with older people being less likely to report ownership and confidence in using them. Older people were also significantly less confident in using computers, with a comparable, nonsignificant pattern for frequency of phone and computer use. Similarly, in line with research identifying digital exclusion in relation to ethnicity, Black people reported significantly less computer access and smartphone and computer confidence compared with the White and other minoritized ethnic groups. However, paranoia severity did not have a significant impact on digital literacy, albeit the high paranoia group skewed toward lower ratings for all variables apart from smartphone confidence. Women were less confident in using computers, and although not reaching significance, computer use and smartphone confidence were also relatively lower for women compared with men.

**Table 1:** Smartphone and computer access, use, and confidence in the SlowMo therapy group (n=168).

Variable	Location				Total
	Sussex (n=57)	Oxford (n=48)	London (n=63)		
Smartphone ownership reported^a^, n (%)	44 (77.2)	30 (76.9)	48 (77.4)	122 (77.2)	
Computer access reported^b^, n (%)	42 (77.8)	26 (66.7)	34 (56.7)	102 (66.7)	
Smartphone use, mean (SD)	63 (37)	61 (38)	57 (35)	60 (36)	
Smartphone confidence, mean (SD)	65 (32)	62 (31)	55 (36)	60 (33)	
Computer use, mean (SD)	51 (38)	46 (34)	43 (33)	47 (35)	
Computer confidence, mean (SD)	63 (32)	57 (26)	50 (32)	56 (31)	

^a^n=158, 93% completion.

^b^n=153, 91% completion.

### Research Question 2: Engagement—Self-reported and System Analytics Adherence to the SlowMo Mobile App

**Figure 3:**
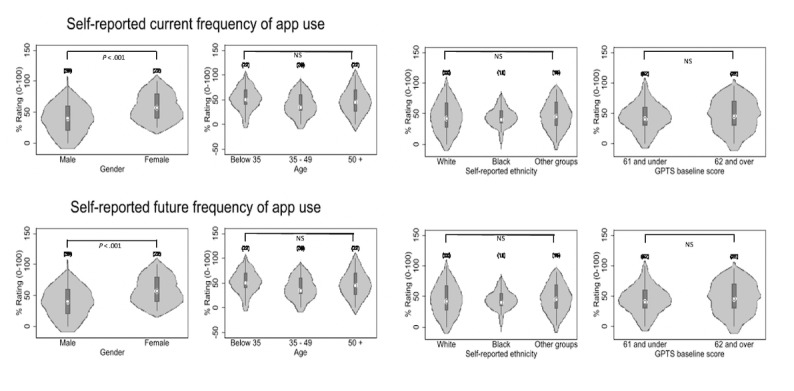
Self-reported current frequency of app use and self-reported future frequency of app use as measures of adherence for participants who completed SlowMo therapy and a user experience assessment (n=82). GPTS: Green Paranoid Thoughts Scale; NS: nonsignificant.

Self-reported current and intended future use of the mobile app are reported in Table S2 in Multimedia Appendix 2. The data indicate that rates of current use varied from “never” to “all of the time,” with participants on average reporting using the app just under half of the time (mean 44.77, SD 25.69). All participants reported at least some intention to use the app again in the future, and average frequency of intended use was also higher than current use, at just over half of the time (mean 62.19, SD 23.00). Self-reported adherence was compared by participant characteristics of age, gender, ethnicity, and paranoia severity, as shown in Figure 3 and Table S3 in Multimedia Appendix 2. Women reported significantly more current and future intended use of the app than men. There were no significant differences in current and intended use for age, ethnicity, or paranoia severity.

For system analytics adherence, 65.4% (100/153) of participants in the therapy group met the mobile app criterion. This increased to 71.4% (100/140) for participants who attended at least 1 session (and were therefore provided with a mobile phone with the mobile app installed). In the subgroup who attended all 8 sessions, this increased further to 80.7% (96/119), suggesting a high rate of adherence. One-fifth of participants (26/119, 21.8%) used the mobile app outside of every recorded session. System analytics adherence was compared for demographic factors and pretherapy smartphone use and confidence, as shown in Table 2. There were no significant differences in analytics adherence to the mobile app for age, gender, ethnicity, or paranoia severity. However, adherence in people who attended all 8 sessions was associated with using smartphones more frequently and being more confident in their use prior to therapy.

**Table 2:** System analytics of adherence to the mobile app compared by age, gender, ethnicity, paranoia severity, and smartphone digital literacy (n=140).

Participant variable	Attended at least 1 session				Attended all 8 sessions		
	Test value	*P* value	MD^a^ CI	Test value		*P* value	MD CI
Age	*χ*^2^_2_=4.65	.10	N/A^b^	*χ*^2^_2_=2.32		.31	N/A
Gender	*χ*^2^_1_=1.01	.32	N/A	*χ*^2^_1_=0.65		.42	N/A
Ethnicity	*χ*^2^_2_=0.19	.55	N/A	*χ*^2^_2_=0.96		.86	N/A
Paranoia severity	*χ*^2^_1_=0.37	.54	N/A	*χ*^2^_1_=0.01		.95	N/A
Smartphone use—frequency	t_101_=–1.17	.24	–27.49 to 7.07	t_90_=–2.48		.02	–46.33 to –5.13
Smartphone use—confidence	t_124_=–1.58	.12	–23.19 to 2.55	t_108_=–2.17		.03	–32.16 to –1.45

^a^MD: mean difference.

^b^N/A: not applicable.

### Research Question 3: Engagement—UES for the SlowMo Mobile App

**Figure 4:**
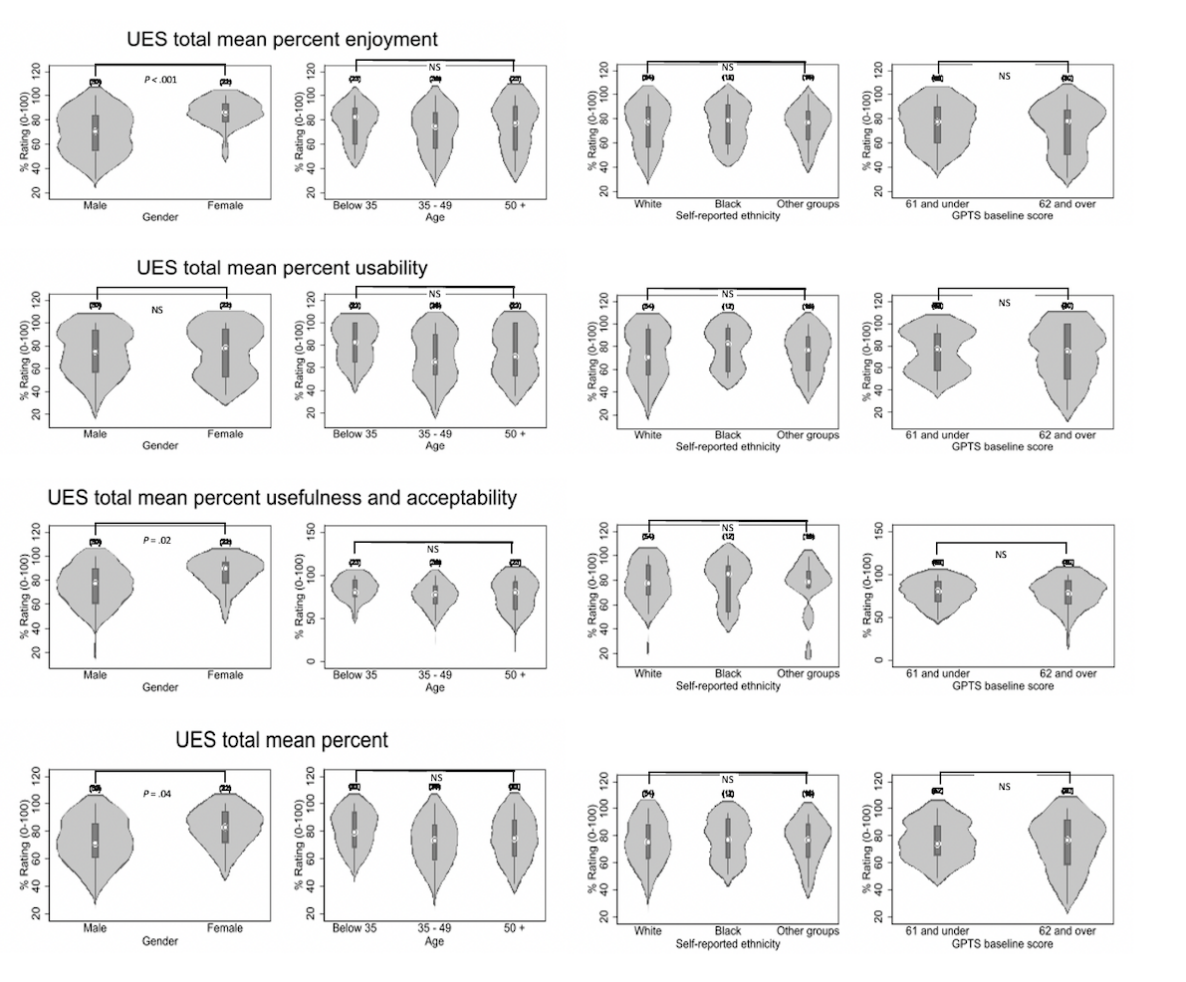
Mean scores on the user experience survey (UES) subscales of enjoyment, usability, usefulness, and acceptability as well as total scores by gender, age, ethnicity, and paranoia severity in participants who completed SlowMo therapy and a user experience assessment (n=82). GPTS: Green Paranoid Thoughts Scale; NS: nonsignificant.

The UES findings for each subscale and the total score are presented in Table S4 in Multimedia Appendix 2. UES ratings were comparable across all subscales, with the majority of people providing positive ratings for enjoyment, usability, and usefulness (overall score: mean 75%, SD 17.06%). However, there was a large range of scores, suggesting that the mobile app was positively received by most, but not all, participants. The UES ratings were compared by demographics (see Figure 4 and Table S5 in Multimedia Appendix 2). There were significant differences for gender, with women reporting higher rates of enjoyment and usefulness, although rates of usability were similar for men and women. Significant differences in digital literacy prior to therapy did not appear to generalize to self-reported user experience, as there were no significant differences for age and ethnicity. There were also no differences in UES ratings in relation to paranoia severity.

## Discussion

### Principal Findings

In this study, the presence of the “digital divide” was replicated in relation to age and ethnicity, with older people and Black people reporting reduced phone or computer access and less confidence in using both types of technology. This is consistent with previous studies of psychosis [6] and the general population [2], indicating higher rates of digital exclusion in relation to age and ethnicity. Women were also found to be less confident in using computers compared with men. Previous evidence is equivocal in relation to gender, with indications of female exclusion in the general population and an absence of gender effects or male exclusion being more common in mental health samples [2,29]. Although we did not find an association between paranoia severity and digital literacy, in contrast to previous findings indicating higher rates of exclusion in mental illness, this may be due to sample characteristics, as a schizophrenia-spectrum diagnosis was an inclusion criterion for the SlowMo trial. Alongside the between-group effects, it is notable that the digital literacy variables often had multimodal distributions, suggesting marked variability in technological competencies. This underscores the need for an individual assessment of digital literacy, regardless of a person’s demographic background, to identify needs and support engagement.

The results further indicated high rates of engagement with the SlowMo therapy mobile app, based on a multimodal assessment incorporating behavioral and experiential measures. The a priori criterion for mobile app adherence was met by 81% of participants who completed all 8 sessions. This is of note, given that prompts were not provided as mandatory nor was use incentivized as part of the trial design, in contrast to other research investigating mobile apps for psychosis [30-32]. We have also previously reported high rates of engagement with therapy sessions (80%) and the SlowMo web app session content (95%) [5,33]. Killikelly et al [34] reviewed adherence in 20 studies of digital therapeutics for psychosis and found that adherence ranged from 28% to 100%, with a mean of 83%, suggesting that adherence to the SlowMo mobile app and web app was consistent with previous research. This is notable, as our sample (n=140) was markedly larger than the included studies (70% sampled, n<40, range=9-104). Further, the experiential assessment of engagement strengthened the conclusions from the behavioral measurement, as the self-report user experience ratings suggest that most people perceived the mobile app as easy to use, enjoyable, and useful.

Importantly, age, ethnicity, and paranoia severity were not associated with any behavioral or experiential engagement measures, in line with our previous findings that these variables also did not moderate therapy outcomes [5]. This suggests that the SlowMo therapy design was effective at bridging the “digital divide,” as demographic differences in digital literacy at baseline did not generalize to differences in engagement during therapy. Unsurprisingly, people who reported being more confident, frequent users of smartphones prior to starting therapy were more likely to be adherent to the mobile app. As mentioned previously, this insight reinforces the importance of digital literacy assessments so that individualized technical support can be provided as needed. We also found that women were significantly more likely than men to report current and future adherence to the mobile app and higher rates of usefulness and enjoyment, although usability ratings and adherence assessed by analytics were comparable. This is consistent with previous findings that women in the general population and those with psychosis are more likely to engage in digital therapeutics than men [35,36]. We tentatively suggest that a key obstacle to men’s engagement with the mobile app may have been due to the home screen displaying users’ worries, which is inconsistent with design research insights that, on average, men prefer solution-orientated approaches [36]. Accordingly, we plan to modify the interface to focus primarily on safer thoughts and strategies, with additional interactions required to access upsetting thoughts, and will test if this does optimize the user experience for a diverse range of men.

Overall, the study results suggest that the SlowMo therapy design did enhance the user experience as intended for a diverse range of people and therefore shows promise in overcoming well-documented challenges to engagement. Our previous inclusive, human-centered design research highlighted the need for psychosis digital therapeutics to be usable, trustworthy, enjoyable, personalized, and normalizing and to offer flexible interpersonal support [19]. This shaped the SlowMo therapy design, such as the use of personalized bubbles as a simple visual metaphor to represent thoughts and coding a native app to support privacy. We therefore recommend adopting an inclusive, human-centered design approach in the development of digital therapeutics. This includes applying design thinking methodology and critically, ensuring purposive sampling from a wide range of people, to support co-design that is representative of the target population.

The findings are also in line with the framework by Perski et al [3] for factors influencing engagement with digital therapeutics, which, as mentioned previously, demonstrates how an individual’s sociocultural context may influence digital therapeutic engagement. In support of this framework, we found demographic differences in digital literacy, which appeared to be attenuated by the SlowMo therapy design, as these differences did not generalize to behavioral or experiential engagement. Consistent with findings of lower digital literacy in women [2,29], we found men were more confident in using technology at baseline. Conversely, women reported more use and satisfaction with the mobile app, in line with findings indicating that women engage better with health apps [35]. Our findings build on the framework by Perski et al [3] by highlighting how therapeutic design interacts with population characteristics to determine engagement, although this hypothesis requires more rigorous research with experimental manipulation of intervention designs in different demographic groups. A limitation of the work is that at least some mobile app analytics were lost for 28 people in the therapy sample due to a bug in the code, although we do not consider that these analytics data likely differed from the rest of the sample. Future work will allow us to validate our findings with larger samples.

### Clinical Implications

Digital therapeutics need value propositions of delivering clinically meaningful outcomes for a wide range of people, given that most health technologies fail to be adopted, scaled up, spread, and sustained, even where they are efficacious in randomized controlled trials [37]. The tailoring of the SlowMo design to its specific target problem, a range of intended users, and the delivery context, as evidenced by the bridging of the “digital divide,” supports initial adoption. We are currently refining an implementation strategy for SlowMo, incorporating the learning from this study. Given the impact of SlowMo on a range of outcomes including well-being, we plan to build on this by incorporating other therapeutic targets and techniques, using principles of agile science and responsive technology [38,39]. This study suggests that inclusive, human-centered design should be incorporated in the design of digital therapeutics, to increase the likelihood they are fit for purpose “in the wild.”

### Conclusion

In conclusion, the findings suggest that the SlowMo therapy trial sample experienced a “digital divide” with a lack of technology access, confidence, or use associated with age, ethnicity, and gender that was consistent with previous research indicating digital exclusion in those who are older, are female, or are from a minoritized ethnic group [2,3]. Experiential and behavioral measures of engagement however found that these differences did not generalize to the user experience of the SlowMo mobile app for age and ethnicity. Self-reported user experience was higher in women, consistent with findings of women engaging more with health apps [35]. The study validates our previous design research [19], as it suggests the SlowMo design optimized the user experience of the intervention as intended and resulted in high rates of adherence for a diverse range of people. This study is in line with a recent co-produced call for digital therapeutic research to focus on how we can enhance existing interventions, the impact of psychosis on engagement, and whether digital therapies can improve reach and access for minoritized groups [40]. Together with the clinical efficacy and moderation results from the SlowMo trial, the findings support the further development of SlowMo therapy and testing in routine services. Our approach underscores the need to focus on user experience as a means of optimizing effectiveness when developing therapeutics, and we strongly advocate the adoption of this strategy to improve outcomes in mental health care.

## Appendix

Multimedia Appendix 1 User experience survey (UES).

Multimedia Appendix 2 Supplementary tables.

## Abbreviations

CBT: cognitive behavioral therapy
GPTS: Green Paranoid Thoughts Scale
NHS: National Health Service
NIHR: National Institute for Health Research
TAU: treatment as usual
UES: user experience survey
