# Supplementary material for: Bridging the Digital Divide in Psychological Therapies: Observational Study of Engagement With the SlowMo Mobile App for Paranoia in Psychosis
Source: JMIR Hum Factors. 2022 Jul 1;9(3):e29725. doi: 10.2196/29725 (PMC9288106; doi:10.2196/29725)
Supplement: Multimedia Appendix 1 [file humanfactors_v9i3e29725_app1.docx]

## **User Experience Survey**

We are interested in your experience of using the SlowMo mobile app. Your feedback is very important for helping us to improve the app, so please be as honest as possible.

Please rate each of the items below on a scale of 0 = ‘totally disagree’ to 10 = ‘totally agree’.

Rating

(0 – 10)

1. I enjoyed using the app ­­­­­­­­­_______
2. The app was too complicated to be helpful to me ­­­­­­­­­_______
3. The app was easy to use ­­­­­­­­­_______
4. The app was boring ­­­­­­­­­_______
5. The app helped me to manage my difficulties better ­­­­­­­­­_______
6. I would recommend the app to people with similar difficulties to me ­­­­­­­­­_______
7. I couldn’t get used to the app ­­­­­­­­­_______
8. The app felt relevant to me and my problems ­­­­­­­­­_______
9. I felt frustrated using the app ­­­­­­­­­_______
10. The information on the app was easy to understand ­­­­­­­­­_______
11. The app was fun ­­­­­­­­­_______
12. The app was not useful for managing my problems ­­­­­­­­­_______

1, 4*, 9*, 11 - enjoyment

2*, 3, 7, 11 - usability

5, 6, 8, 12* - usefulness/acceptability

* reverse score
