# Supplementary material for: Bridging the Digital Divide in Psychological Therapies: Observational Study of Engagement With the SlowMo Mobile App for Paranoia in Psychosis
Source: JMIR Hum Factors. 2022 Jul 1;9(3):e29725. doi: 10.2196/29725 (PMC9288106; doi:10.2196/29725)
Supplement: Multimedia Appendix 2 [file humanfactors_v9i3e29725_app2.docx]

Table S1. Digital literacy in the SlowMo therapy group who attended at least one session compared by age, gender, ethnicity and paranoia severity (n = 168).

| Digital literacy variable | Participant  variable | Test (df) | Value | p | MD CI |
| --- | --- | --- | --- | --- | --- |
| Smartphone ownership | Age | x^2^(2) | 23.11 | <.001** | - |
|  | Gender | x^2^(1) | 0.03 | .853 | - |
|  | Ethnicity | x^2^(2) | 3.23 | .199 | - |
|  | Paranoia severity | x^2^(1) | 0.01 | .942 | - |
| Frequency of smartphone use | Age | F(2,124) | 2.50 | .086 | - |
|  | Gender | t(125) | 0.41 | .683 | 11.55,17.59 |
|  | Ethnicity | F(2,124) | 2.14 | .122 | - |
|  | Paranoia severity | t(125) | -0.10 | .920 | 13.80,12.47 |
| Confidence in smartphone use | Age | F(2,154) | 14.10 | <.001** | - |
|  | Gender | t(155) | -1.06 | .293 | -18.21,5.53 |
|  | Ethnicity | F(2,154) | 3.74 | .026* | - |
|  | Paranoia severity | t(155) | 0.18 | .855 | -9.73,11.73 |
| Computer  access | Age | x^2^(2) | 3.56 | .168 | - |
|  | Gender | x^2^(1) | 0.59 | .442 | - |
|  | Ethnicity | x^2^(2) | 7.44 | .024* | - |
|  | Paranoia severity | x^2^(1) | 0.05 | .815 | - |
| Frequency of computer use | Age | F(2,122) | 2.89 | .059 | - |
|  | Gender | t(76) | -1.75 | .085 | -23.55,1.54 |
|  | Ethnicity | F(2,122) | 0.49 | .614 | - |
|  | Paranoia severity | t(123) | 0.49 | .628 | -9.64,15.92 |
| Confidence in computer use | Age | F(2,154) | 13.08 | <.001** | - |
|  | Gender | t(155) | -2.30 | .023* | -23.40,-1.80 |
|  | Ethnicity | F(2,154) | 4.86 | .009** | - |
|  | Paranoia severity | t(104) | 0.51 | .615 | -8.01-13.48 |

* p < .05

** p < .005

Table S2. Self-reported current and future usage of the SlowMo mobile app in people who completed SlowMo therapy and a user experience assessment, compared by age, gender, ethnicity and paranoia severity (n = 82).

|  | Mean (SD) | Range |
| --- | --- | --- |
| How much have you been using the app? | | |
| Sussex (n = 28) | 37.07 (25.94) | 0 - 90 |
| Oxford (n = 15) | 55.33 (25.67) | 0 - 100 |
| London (n = 37) | 46.32 (24.35) | 0 - 100 |
| Total (n = 80) | 44.77 (25.69) | 0 - 100 |
| Will I use the app in the future? | | |
| Sussex (n= 26) | 56.96 (23.14) | 10 - 100 |
| Oxford (n = 15) | 68.20 (24.42) | 30 - 100 |
| London (n = 37) | 63.43 (22.17) | 10 - 100 |
| Total (n = 78) | 62.19 (23.00) | 10 - 100 |

Table S3. Self-reported current and intended future use of mobile app in people who completed SlowMo therapy and a user experience assessment, compared by age, gender, ethnicity and paranoia severity (n = 82).

| Self-reported adherence  variable | Participant variable | Test(df) | Value | p | Mean difference confidence interval |
| --- | --- | --- | --- | --- | --- |
| Self-reported current use of mobile app | Age | F(2,77) | 1.53 | .222 | - |
|  | Gender | t(78) | -3.26 | .002** | -31.9,-7.72 |
|  | Ethnicity | F(2,77) | 0.04 | .957 | - |
|  | Paranoia | t(78) | -0.99 | .325 | -17.82,5.97 |
| Self-reported intended future use of mobile app | Age | F(2,75 | 1.13 | .327 | - |
|  | Gender | t(76) | -2.99 | .004** | -27.48,-5.53 |
|  | Ethnicity | F(2,75) | 0.55 | .582 | - |
|  | Paranoia | t(76) | -0.43 | .669 | -13.11,8.46 |

* p < .05

** p < .005

Table S4. User Experience Survey of mobile app in people who completed SlowMo therapy and a user experience assessment, compared by age, gender, ethnicity and paranoia severity (n = 82).

| UES variable |  | Sussex | Oxford | London | Total |
| --- | --- | --- | --- | --- | --- |
| Enjoyment | Mean % (SD) | 72.08 (17.70) | 73.32 (20.32) | 75.19 (19.04) | 73.75 (18.64) |
|  | Range | 31 – 98 | 45 - 100 | 35 - 100 | 31 – 100 |
| Usability | Mean % (SD) | 73.71 (19.73) | 74.32 (24.12) | 73.86 (22.12) | 73.89 (21.42) |
|  | Range | 33 – 100 | 23 - 100 | 35 – 100 | 23 – 100 |
| Usefulness | Mean % (SD) | 76.32 (17.80) | 79.10 (14.43) | 77.44 (19.11) | 77.35 (17.70) |
|  | Range | 25 - 100 | 60 - 100 | 20 - 100 | 20 – 100 |
| Total UES | Mean % (SD) | 74.03 (16.84) | 75.58 (17.61) | 75.50 (17.44) | 74.99 (17.06) |
|  | Range | 30 - 99 | 46 - 100 | 42 - 100 | 30 – 100 |

Table S5. User experience of mobile app in SlowMo therapy completers compared by age, gender, ethnicity and paranoia severity (n = 82).

| User experience variable | Demographic variable | Test (df) | Value | p | Mean difference confidence interval |
| --- | --- | --- | --- | --- | --- |
| Enjoyment | Age | F(2,79) | 0.58 | .588 | - |
|  | Gender | t(80) | -3.52 | .001** | -23.00,-6.00 |
|  | Ethnicity | F(2,79) | 0.53 | .949 | - |
|  | Paranoia | t(80) | 1.01 | .315 | -4.54,13.18 |
| Usability | Age | F(2,79) | 2.15 | .123 | - |
|  | Gender | t(80) | -0.16 | .875 | -12.05,9.84 |
|  | Ethnicity | F(2,79) | 0.28 | .754 | - |
|  | Paranoia | t(80) | 1.79 | .078 | -1.00-18.30 |
| Usefulness | Age | F(2,79) | 1.26 | .290 | - |
|  | Gender | t(80) | 2.45 | .016* | -19.02,-2.98 |
|  | Ethnicity | F(2,79) | 0.16 | .855 | - |
|  | Paranoia | t(80) | 0.75 | .456 | -5.1,11.14 |
| Total UES | Age | F(2,79) | 1.58 | .212 | - |
|  | Gender | t(80) | -2.14 | .036* | -17.17,-1.15 |
|  | Ethnicity | F(2,79) | 0.05 | .956 | - |
|  | Paranoia | t(80) | 1.37 | .174 | -2.40,13.08 |

* p < .05

** p < .005
